# Slide electrification of drops at low velocities†

**DOI:** 10.1039/d4sm00019f

**Published:** 2024-03-19

**Authors:** Chirag Hinduja, Hans-Jürgen Butt, Rüdiger Berger

**Affiliations:** a Max Planck Institute for Polymer Research 55128 Mainz Germany berger@mpip-mainz.mpg.de

## Abstract

Slide electrification of drops is mostly investigated on tilted plate setups. Hence, the drop charging at low sliding velocity remains unclear. We overcome the limitations by developing an electro drop friction force instrument (eDoFFI). Using eDoFFI, we investigate slide electrification at the onset of drop sliding and at low sliding velocities ≤ 1 cm s^−1^. The novelty of eDoFFI is the simultaneous measurements of the drop discharging current and the friction force acting on the drop. The eDoFFI tool facilitates control on drop length and width using differently shaped rings. Hereby, slide electrification experiments with the defined drop length-to-width ratios >1 and <1 are realized. We find that width of the drop is the main geometrical parameter which determines drop discharging current and charge separation. We combine Kawasaki–Furmidge friction force equation with our finding on drop discharging current. This combination facilitates the direct measurement of surface charge density (*σ*) deposited behind the drop. We calculate *σ* ≈ 45 μC m^−2^ on Trichloro(1*H*,1*H*,2*H*,2*H*-perfluorooctyl)silane (PFOTS) and ≈20 μC m^−2^ on Trichloro(octyl)silane (OTS) coated glass surfaces. We find that the charge separation by moving drops is independent of sliding velocity ≤ 1 cm s^−1^. The reverse sliding of drop along the same scanline facilitates calculation of the surface neutralization time constant. The eDoFFI links two scientific communities: one which focuses on the friction forces and one which focuses on the slide electrification of drops.

## Abstract 2

The novelty of our electro drop friction force instrument (eDoFFI) is the simultaneous measurements of the drop discharging current and friction force acting on the sliding drops. With eDOFFI, we measure the surface charge density deposited behind sliding drops.

## Graphical abstract

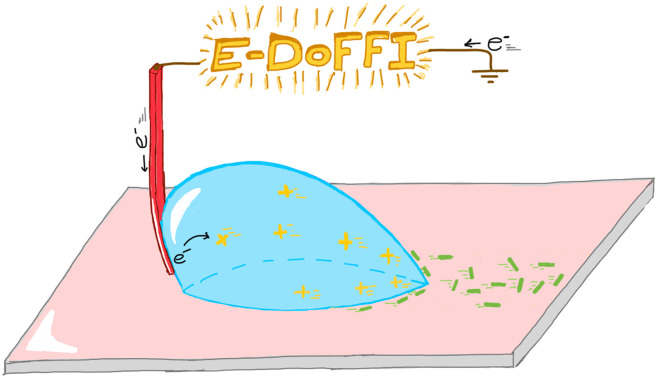

## Introduction

Water drops sliding down on plant leaves or hydrophobic surfaces get electrically charged.^1–4^ This phenomena is known as slide electrification. Understanding and controlling drop sliding behavior is the key knowledge in dropwise condensation,^5^ inkjet printing,^6^ water desalination,^7^ and drop manipulation in microfluidic devices.^8^ Slide electrification studies are mostly focused on drops falling on surfaces and subsequently sliding down on a tilted plane.^9–14^ Here we investigate slide electrification at the onset of drop sliding and at low sliding velocities ≤ 1 cm s^−1^. These two conditions are not easily accessible on tilted plane slide electrification measurements, but are required for detailed understanding slide electrification of drops.

On a tilted plate setup, a drop, at first, slides down on a neutral surface. Once the drop has traversed a certain distance, it is discharged through an electrode to quantify the charge acquired during its descent.^12^ In tilted plate experiments, the drop velocity continuously increases due to the influence of gravity. As a result, the drop reaches velocity of typically 0.1 m s^−1^ depending on the drop mass and the plate's tilt angle.^15–17^ At such drop velocities, the charge separation decreases with increasing drop velocities.^18^ However, the inclined plane possess a drawback: due to gravity, it becomes impractical to control the drop velocity and attain slowly sliding drops. As a result, less is known about the charge separation at the onset of sliding and low sliding velocities - in the order of mm s^−1^. For drop velocities accessible on the tilted plane, the hydrodynamic dissipation and inertial effects play an important role. Due to these effects, the shape of the drop's base area changes from oval at the onset to cusp/pearling at high capillary numbers.^15,16^ As a result, it becomes impractical to control drop foot print shape on the inclined plane. Therefore, it is unknown which geometrical parameter influences drop discharging the most.

**Fig. 1:**
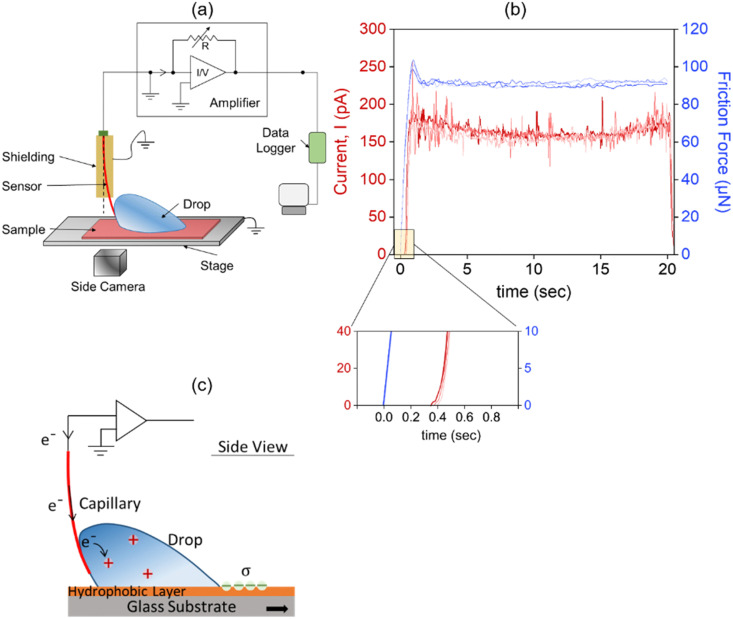
(a) A schematic setup representing simultaneous measurements of the discharging current and the friction force acting on a sliding drop. The setup involves an electrically conductive capillary, which holds the drop in position when the stage moves, an amplifier which augments the discharging current signal, and a side camera which tracks the displacement of conductive capillary and changes in dynamic contact angles of the drop. (b) Friction force and current signals for a 7 μL Milli-Q water drop (resistivity 18.2 MΩ cm at 25 °C) from the onset of sliding to the kinetic regime of drop sliding at a stage speed of 2 mm s^−1^. The signals are obtained for the drops sliding along three different scan-lines on a glass substrate coated with a hydrophobic layer. For each scan line we used a fresh water drop. (c) A schematic in side view describing the electrical current flow in eDoFFI.

To overcome limitations posed by the tilted plate setup, we have developed an in-house electro drop friction force instrument (eDoFFI). The novelty of eDoFFI is simultaneous measurements of drop discharging current and friction force acting on a sliding drop. It involves a gold coated-conductive glass capillary sensor which acts both as a force sensor and a current collector. The friction force is measured by quantifying the deflection of the sensor using side camera and the current signal is acquired by a low noise transimpedance amplifier (Fig. 1a). The eDoFFI allows us to move the drops at constant speeds which corresponds to low capillary numbers. For a water drop at 20 °C, the capillary number 
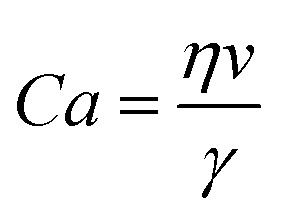
 is in the range 10^−6^ ≤ Ca≤ 10^−4^. Where, *η* is dynamic viscosity, *v* is drop speed, and *γ* is the surface tension of the liquid. In this article, we address two questions using eDoFFI. First: How does drop velocity influence the drop discharging current and charge separation? Second: Which drop geometrical parameter influences charge separation the most?

## Results and discussion

The friction force acting on a drop at the onset of sliding and at slow speeds is described by Kawasaki and Furmidge (eqn (1)).^19–26^

1 *F* = *k*·*γ*·*w*·(cos *θ*_r_ − cos *θ*_a_)

here, *k* is a geometrical factor, *γ* is surface tension of the liquid, *w* is the width of the contact area of the sliding drop, and *θ*_r_ and *θ*_a_ are the receding and advancing contact angles of the drop, respectively. This friction force acting on a drop is quantified using our drop friction force instrument (DoFFI).^27–30^ In drop friction measurements, a drop is attached to the capillary and the stage underneath moves. As a result of the stage motion, the glass capillary deflects. This deflection is quantified by a camera from the side view. Multiplying this deflection with the spring constant of the capillary provides us with the friction force. We perform investigations on a 7 μL Milli-Q water drop displaced on trichloro(1*H*,1*H*,2*H*,2*H*-perfluorooctyl)silane (PFOTS) coated glass substrates. The drop is immobilized by the conductive capillary while the stage underneath moves at 2 mm s^−1^. This stage speed corresponds to a Ca ≈ 3 × 10^−5^. The friction force is zero when the stage is at rest (blue data in Fig. 1b). As the stage moves, the capillary starts to deflect corresponding to an increase in friction force (blue curves Fig. 1b inset). The side view image acquisition and analysis indicates that, at the onset of drop sliding, the receding contact line displaces ≈0.3 s after the advancing contact line starts (advancing and receding contact line speeds in Fig. S1, ESI†). Therefore, until ≈0.3 s the friction force values emanate from the motion of the advancing contact line. After 0.3 s, advancing and receding contact lines move at different velocities and constitute to the further increase in friction force. The friction force exhibits a maxima of 100 μN when both the advancing and receding contact line velocities become equal to 2 mm s^−1^ (ESI,† Fig. S2). Thereafter, the force decreases to a constant value of ≈90 μN in the kinetic regime.

In addition to friction measurement, the conductive capillary measures the drop's discharging current. The friction force and current are acquired simultaneously. Similar to friction force, the current is zero when the drop is at rest. However, contrary to friction, the current signal continues to remain zero when only the advancing contact line displaces. Once the receding contact line displaces, that is at ≈0.3 s, an increasing current signal is observed (red curves Fig. 1b inset). At this point, the electric double layer breaks and the charges are separated at the receding edge (Fig. 1c). At hydrophobic surfaces, the negative charges at the solid–liquid interface are most likely due to an enrichment of hydroxyl anions.^31–36^ While negative ions (OH^−^) are adsorbed at the solid–air surface, hydronium ions (H^+^) ions stay in the drop. The drop is connected to the conductive capillary which is then connected to the ground *via* an amplifier. Therefore, these hydronium ions (H^+^) are neutralized by the flow of electrons from the ground to the drop (Fig. 1c). This flow of electrons is recorded as drop discharging current *via* the amplifier. The drop discharging current is the average of the total charge separated along the entire receding contact line length.

Similar to friction, the current reaches a maximum (individual current profiles are provided in the ESI,† Fig. S3). The appearance of a maximum in the current signal indicates the possibility of current dependency on the drop width or drop footprint area or receding contact line length. Once the contact line acquires a defined shape, we measure a constant current ≈170 pA in the kinetic regime (Fig. 1b). Slight variations in localized surface chemistry variations result in a local change in current signal over the slide length in the kinetic regime. We estimate the total charge separated due to drop sliding by integrating the discharging current over the time of drop motion. For a 7 μL water drop sliding over the distance of 40 mm, we estimate a drop charge of 3 ± 0.5 nC. Thus, eDoFFI facilitates both friction and real-time current measurement in low Ca regime of drop sliding. We find that drop charging follows the analogy of drop friction. That is, it can be classified into sub categories of static, transient and kinetic discharging current profiles similar to drop friction.^37^

Next, we explore which drop parameter influences charge separation and the discharging current. Can we derive an expression for the discharging current similar to the friction force (eqn (1))?

**Fig. 2:**
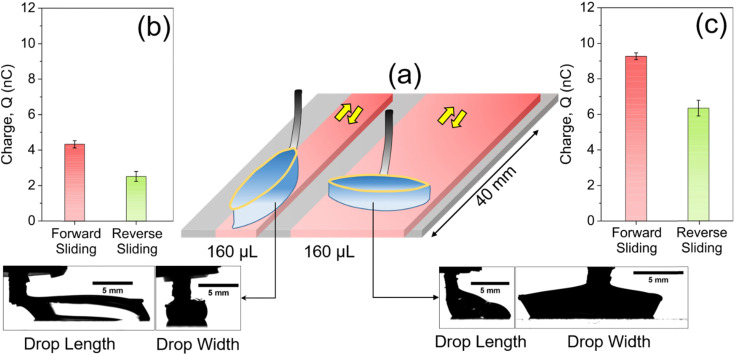
(a) Sketch of the elliptical rings (orange) attached to the capillaries (grey). The elliptical ring dictates the drop's foot print area. The ring, which results in lengthier drop relative to the sliding direction (left side) has a major axis of 14.3 ± 0.1 mm and a minor axis of 4.8 ± 0.1 mm. The ring resulting in wider drops relative to the sliding direction (right side) has a major axis of 14 ± 0.1 mm and a minor axis of 4.2 ± 0.1 mm. The corresponding contour images in side and front view are recorded with a drop of 160 μL moving at a speed of 2 mm s^−1^ over a length of 40 mm on a PFOTS/glass surface. Scale bars in the images are 5 mm. The ring to surface height is 2.2 mm. (b) The calculated charge values for drops having length-to-width ratio more than 1. The charge is calculated by integrating the current profiles over a sliding length of 40 mm. Forward sliding: when the drop slides on a neutral surface (red bar). Reverse sliding: when the drop slides back along the same path after 2 s of waiting time (green bar). (c) The charge values for the drops having length-to-width ratio of less than 1. Error bars represent variation across 3 scan lines on a sample.

The e-DoFFI tool facilitates us to control the length, the width, and the foot print area of the drop. The control in drop shape is achieved by employing elliptically shaped silver wires at the end of the capillary (Fig. 2a). These shaped rings change the drop length and width, and renders the drop base possibly to an ellipse. We prepare the longest and widest drops using two different elliptical rings in order to probe the dependency of charge separation on different geometries of drops. First, we slide a 160 μL drop at 2 mm s^−1^ with the elliptical ring having its major axis parallel to the drop sliding direction (left schematic in Fig. 2a). This ring results in a drop length of = 15 ± 0.2 mm and a width of = 4.5 ± 0.2 mm in the kinetic regime. In other words, drop length-to-width ratio is more than 1. For a 40 mm drop sliding distance on a neutral PFOTS/glass surface, we measure a total charge of (4.3 ± 0.5) nC (Forward Sliding Fig. 2b). Next, we perform experiments where the major axis of the elliptical ring is perpendicular to the drop sliding direction (right schematic in Fig. 2a). This elliptical configuration results in a drop length of = 7.7 ± 0.2 mm and a width of = 12 ± 0.4 mm in the kinetic regime. Here, the drop length-to-width ratio is less than 1. For the same drop sliding distance, we measure an increased charge of (9.3 ± 0.5) nC on a neutral PFOTS/glass surface (Forward Sliding Fig. 2c).

To verify the dependency of charge separation on drop geometrical parameters, we normalize the charge value to the respective measured drop width and length. For the ring, which result in longer drop relative in direction of sliding, we estimate ratios of *Q*_1_/*L*_1_ = 0.3 ± 0.1 nC mm^−1^ and *Q*_1_/*w*_1_ = 0.95 ± 0.2 nC mm^−1^. For the ring which, results in wider drops, we estimate ratios of *Q*_2_/*L*_2_ = 1.2 ± 0.2 nC mm^−1^ and *Q*_2_/*w*_2_ = 0.8 ± 0.1 nC mm^−1^. *Q*_1_ and *Q*_2_ are the values of charge, *L*_1_ and *L*_2_ are the drop lengths, *w*_1_, and *w*_2_ are the drop widths for the respective cases of drop sliding. Among the obtained ratios, the charge values normalized with widths (*Q*_1_/*w*_1_,*Q*_2_/*w*_2_) give us an equal ratio. We conclude that the drop charge can be proportional to drop width.

**Fig. 3:**
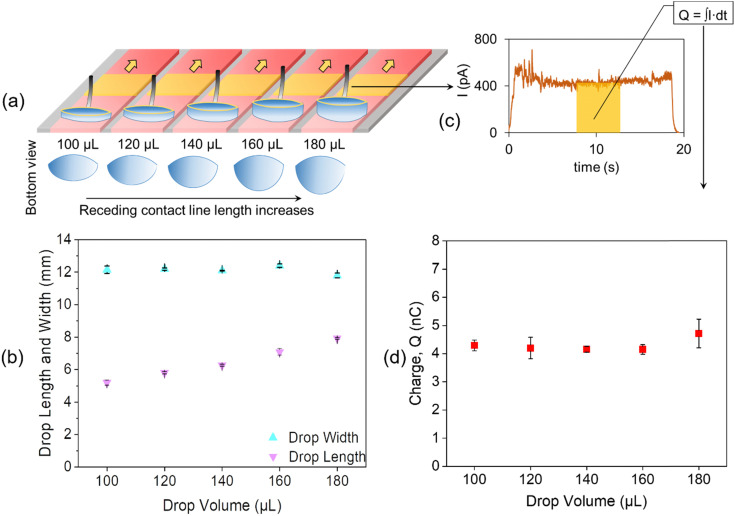
(a) A schematic of different drop shapes for an increasing drop volume. For an increasing volume, we increase the ring to surface height to achieve a constant drop width. In particular, with increasing drop volume, the receding contact line length increases, which we sketched below. (b) Measured drop length and width in the kinetic regime for each drop volume sketched in (a). (c) Representative current profile. The orange highlighted area is considered for the analysis of charge separation across all the drop volumes. (d) The measured charge for drops passing the highlighted area. For each drop volume, a forward sliding motion is performed three times using a fresh drop. The error bars correspond to the 95% confidence level.

The two measurements with differently shaped elliptical rings (Fig. 2a, b, and c) are not sufficient to conclude that the drop width influences slide electrification more than the drop length. To gain further insights, we conduct experiments in which we keep the drop width constant and increase the drop length. The increase in drop length is achieved by varying the drop volume (Fig. 3a). The constant drop width is achieved by altering the ring-to-surface height. We systematically vary the drop volume by keeping the drop width constant. The experiments are performed with a drop volume starting from 100 μL, incremented by 20 μL per drop, until we reach a final drop volume of 180 μL (Fig. 3a). We select the elliptical ring having its major axis perpendicular to the sliding direction. For all the drop volumes, a constant drop width of ≈12.5 mm is achieved by altering the ring to surface distance before the drop starts sliding.^30^

With this set of experiments, we investigate whether it is the length or the width or the length of the receding contact line of the drop that determines the charge separation. With increasing drop volume, the drop length increases from ≈5 mm (100 μL) to ≈8 mm (180 μL), while the width stays nearly constant (Fig. 3b). In the static and transient drop sliding region, drop width and length change significantly, therefore, we select the kinetic current and friction region for our detailed analysis. We integrate the current signal for each drop in the kinetic region from time, *t* = 5 to *t* = 15 s (Fig. 3c). This integration gives us the amount of charge separated when the drop traverses an area of 20 mm × 12.5 mm. For each drop volume, we measure a charge of ≈4 nC (Fig. 3d). The amount of charge separated is the same within the uncertainty bars. We conclude that the width of the drop is the main geometrical parameter which determines the drop discharging current and charge separation rather than the drop's volume, receding contact line length, foot print area or length. This finding leads to the proportionality

2 *I* ∝ *w*

It could be confusing that the charge separation occurs at the receding contact line (Fig. 1) and scales with the drop width rather than the receding contact line length. The azimuthal contact angle distribution follows a 3^rd^ order polynomial on hydrophobic surfaces.^38^ Ratschow *et al.* reported that the charge separation depends on the receding contact angle.^18^ The charge separation decreases with a decreasing receding contact angle. Thus, we anticipate that the azimuthal contact angle distribution possibly leads to a variation of the surface charge density, which is left behind the drop (ESI,† Fig. S4). The latter effect can be there but only plays a secondary role compared to drop width. Mainly, charge separation is proportional to the drop width.

The friction force acting on a 160 μL drop during forward sliding is 810 ± 15 μN for the ring creating wider drops *i.e.*, the ring having major axis perpendicular to the sliding direction. The friction force for the ring which creates lengthier drops during forward motion is 300 ± 10 μN. We attribute the difference in magnitude of force to the change in drop width, which changes the friction force (eqn (1)) and the additional torque on the capillary (ESI;† Fig. S6 and S7).

**Fig. 4:**
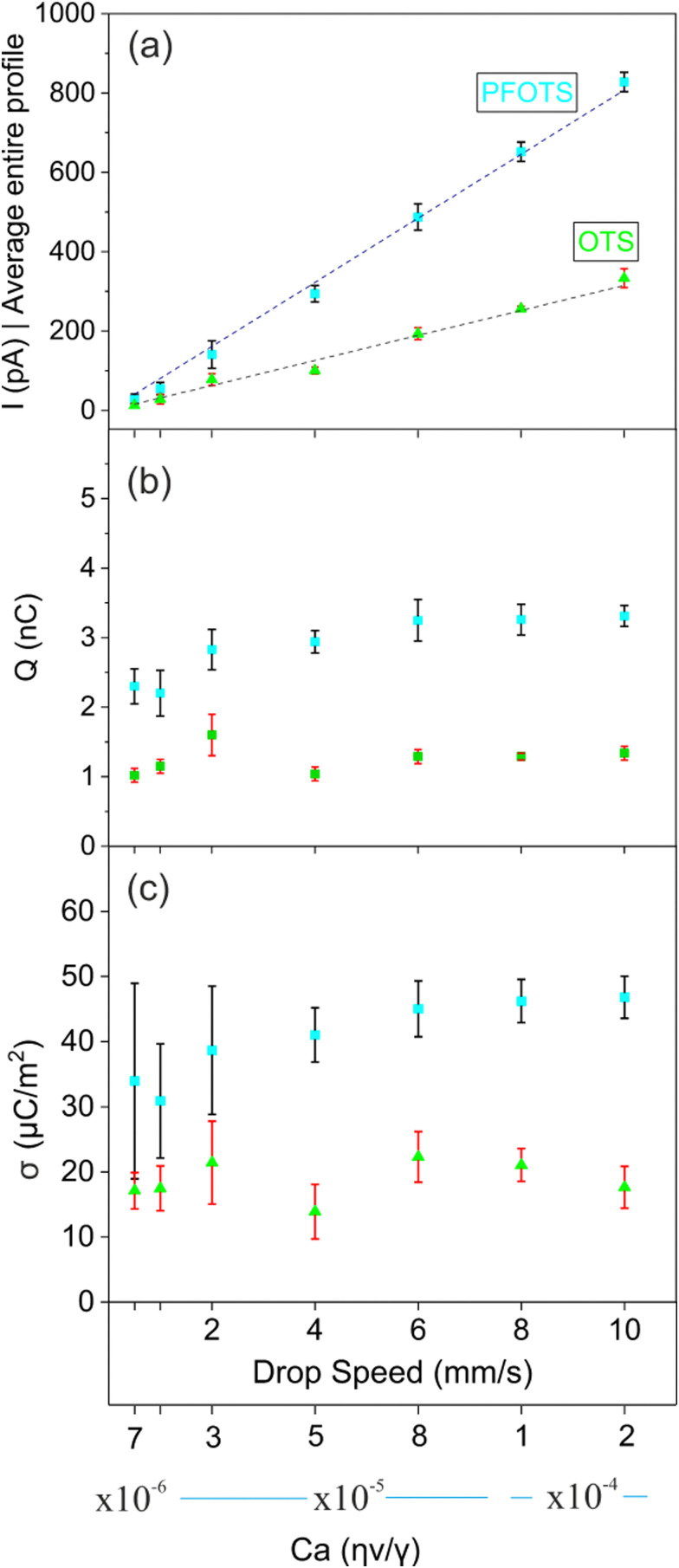
(a) The current averaged over a slide length of 40 mm on OTS/glass sample (green) and on PFOTS/glass sample (cyan). The error bar corresponds to three independent measurements using 5 μL milli-Q water drops for each speed on one sample. The dotted lines are fits by eqn (3). (b) Charge separation over a sliding distance of 40 mm for various speeds on an OTS/glass sample (green) and a PFOTS/glass sample (cyan). The error bar corresponds to three independent measurements for each speed on one sample. (c) Surface charge density calculated using eqn (5) for varying drop speed.

To analyze the drop's speed influence on the charge separation, we slide 5 μL Milli-Q water drops on OTS/glass and PFOTS/glass samples at velocities of 0.5 mm s^−1^ to 10 mm s^−1^ for a total slide length of 40 mm (Fig. 4a). For each velocity, we measure current and friction forces for a fresh drop along three scan lines. With increasing drop speed, the average current value increases linearly for both OTS and PFOTS samples (Fig. 4a). The measured current profiles are numerically integrated to compute the total charge which is separated. The amount of charge separated is found to be independent of the drop speed (Fig. 4b). For the OTS/glass sample, the total amount of charge is within the range of 1–1.5 nC. For the PFOTS/glass sample, we measure a charge of 2–3 nC for the velocities studied here. We conclude that, at low capillary numbers (10^−6^ ≤ Ca ≤ 10^−4^), charge separation is independent of sliding velocity.

For all measurements, we find that the contact line of the drops move smoothly. Contact line pinning is a rare event in our sliding drops experiments. Nonetheless, in the presence of topographic defects, the contact line is first pinned and then upon release, moves at a much faster velocity than the set stage speed.^39^ The latter plays a role in the charge separation. Ratschow *et al.* reported that the charge separation decreases with an increase in the drop velocity.^18^ In particular, they observed that charge separation starts to decrease for a velocity ≥1 cm s^−1^. In our experiments, the stage (or the drop) is moving at a lower speed of 2 mm s^−1^ (or other set speeds). We tracked the receding contact line velocity for the entire sliding length of 40 mm. We found that the receding contact line velocity, including depinning events from the small surface inhomogeneities, always stays below 3 mm s^−1^ (Fig. S1, ESI†). Therefore, we expect that the contact line velocity during depinning does not affect our eDoFFI measurements.

Ratschow *et al.*^18^ report that surface charge density at the receding contact line remains the same for Peclet numbers in the range of 0.001–1. The Peclet number (Pe = *λv*/*α*) is the measure of advective transport of a property relative to its diffuse transport in a flow. Here, *λ* ≈ 100 nm is the Debye length for distilled water, *α* ≈ 10^−9^ m^2^ s^−1^ is the ion diffusivity, and *v* is the drop speed. In our experiments, the velocity corresponds to a Peclet number in the range of 0.1–1. Therefore, our experiments confirm the model proposed by Ratschow *et al.* in the above-mentioned range of Peclet numbers.

Our measurements (Fig. 4a) show that that the drop discharging current is directly proportional to the drop velocity (eqn (3)).

3 *I* ∝ *v*

Combining eqn (2) and (3) the drop discharging current follows

4 *I* = *σ*·*w*·*v*

here *σ* is the average surface charge density behind the drop. This equation is similar to the one hypothesized by Bista *et al.*^40^ There, the authors discharge the drop at the end of sliding on an inclined plane and estimate the change in surface charge density by assuming a constant drop shape throughout the sliding motion. However, in our experiments, the drop is grounded by the capillary during the sliding motion and charges are deposited at the zero drop potential.

The drop length and width change significantly at the onset of sliding^41,42^ and while interacting with topographic and chemical defects.^39,43,44^ Rearranging eqn (1) and (4) allows us to omit drop width from the expression and provides an expression for the surface charge density:5
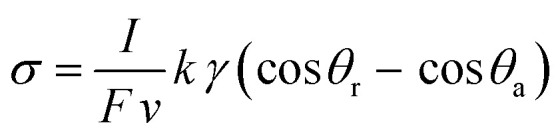
here all the parameters on the right hand side of the equation can be quantified using our in-house developed eDoFFI. The k is a numerical constant and is typically in the range of 0.7–1. For simplicity, we assume *k* = 1 in our calculations. We measure friction force in the order of 85 μN on PFOTS/glass and 50 μN on OTS/glass acting on a 5 μL drop. The sliding advancing and receding contact angles are 120° and 80° on PFOTS/glass surface, 112° and 92° on OTS/glass surface, respectively. Thus, we calculate *σ* ≈ 45 μC m^−2^ on PFOTS/glass and ≈20 μC m^−2^ on OTS/glass for capillary number in the range 10^−6^ ≤ Ca ≤ 10^−4^ (Fig. 4c).

We compare our measured surface charge density with the data available in literature. We compare it with the estimated surface charge density found on PFOTS/glass using inclined plate setup. The samples have been prepared using the same chemical vapor deposition method. The *σ* on PFOTS/glass using eDoFFI is in the same order of magnitude as values of the initial surface charge reported by other authors, *i.e.* 20 μC m^−2^.^12,40^ In fact, the *σ* in our case is ≈10–15% higher compared to values reported. We attribute this systematic higher value of *σ* to a two orders of magnitude smaller drop velocity compared to the experiments performed with the inclined plane. With an increasing drop speed (or Peclet number > 1) the charge separation decreases due to increased advection at receding contact line of the drop.

We term a scan line as “forward” when the drop wets a neutral surface during sliding. We term a scan line “reverse” when the drop slides back along the same scan line. In the experiments plotted in Fig. 2, the waiting time for the drop at the end of forward motion and before start of the reverse motion is about 2 s. For the reverse motion along the same scan line, we observe a decrease in the average current and the total charge value compared to the forward motion. We measure a charge of (2.5 ± 0.3) nC and (6.3 ± 0.5) nC for the smaller and wider drop widths for 40 mm of drop sliding distance, respectively (Fig. 2b and c). The observed charge during reverse motion is ≈40–50% less when compared to forward sliding process. We attribute this decrease in net charge to the already adsorbed negative charges at the surface. Here, in our measurements, the drop is discharged continuously through conductive capillary, therefore, we anticipate that the negative charges are deposited homogeneously along the sliding path during forward motion. Some of these negative charges are annihilated *via* ambient ions and some decay through the glass substrate.^45–50^ Hence, a fraction of negative charges are compensated by the drop's positive charge during its reverse motion. This difference is clearly observed on the magnitude of current profiles (ESI,† Fig. S5). Therefore, during reverse motion, the net charge output decreases. Our observation is consistent to previous reports.^12,51^ For example, Stetten *et al.*^12^ reported that the drop charge on PFOTS/glass surface decreases from ≈1.1 nC for the first drop to ≈0.7 nC for the 2nd drop for an interval of 1.5 s between the drops. This corresponds to nearly 40% decrease in the drop charge. With increasing time intervals between the subsequent drops, the authors reported an increase in drop charge for the 2nd drop. The reason being, more and more surface charges are neutralized with increasing time interval.

The reverse drop sliding facilitates direct calculation of the time constant for the surface neutralization. The current signal during the forward motion, *i.e.* for the drop sliding on a neutral surface follows eqn (4). For the reverse sliding, assuming that the constant charge density is deposited always, the current signal (*I*_rev_) follows eqn (6).6
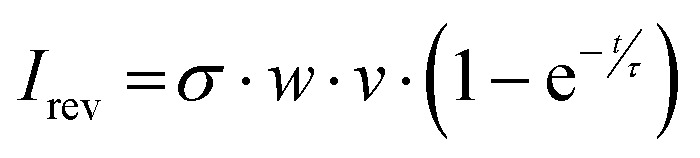
where *τ* is the neutralization time constant, and *t* is the time required by the advancing contact line of the drop during reverse motion to reach the same position as the receding contact line during the forward motion along the scanline (ESI,† Fig. S8). This time *t* includes the drop waiting time between the end of forward motion and the start of the reverse motion. By rearranging eqn (4) and (6), we are able to calculate the time constant for the surface neutralization processes.7
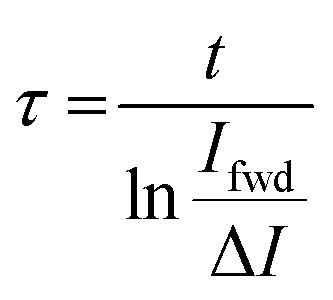
*I*_fwd_ is the discharging current during the forward drop motion. Δ*I = I*_fwd_ − *I*_rev_ is the difference in current signal during the forward and reverse drop motion.

We roughly calculate the neutralization time constants based on the measured average current values. The drops traverse 40 mm distance at 2 mm s^−1^, reaching half distance after 10 s. The waiting time between drop's forward and reverse sliding motion is 2 s. At *t* = 22 s half distance is reached again in the subsequent reverse sliding motion. The average discharging current is ≈220 pA for the forward drop motion, and ≈150 pA for the reverse motion using the ring which results in more drop length. For this drop geometry, we calculate *τ* ≈ 19 s. We obtain a similar value for the drop sliding with the ring resulting in more drop width. Here, the average current during the forward and reverse drop motion correspond to *I*_fwd_ ≈ 500 pA and *I*_rev_ ≈ 350 pA, respectively. Thus, we calculate *τ* ≈ 18 s.

**Fig. 5:**
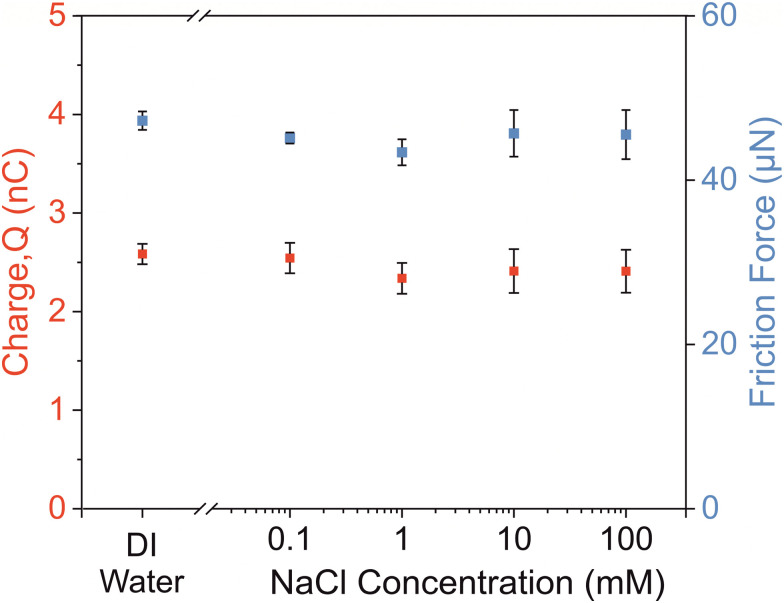
The charge separation (red) and friction force (blue) data for four different NaCl salt concentration drops and deionized (DI) water. Each data point is an average of three independent measurements of 5 μL drops of the respective salt concentration. The data is obtained by sliding the drops at 2 mm s^−1^ velocity over the distance of 40 mm on a PFOTS coated glass sample.

To investigate the influence of salt concentration on charge separation and friction force, we slide 5 μL drops of deionized water and four different sodium chloride (NaCl) solutions. For the drops sliding at 2 mm s^−1^ over the distance of 40 mm, we measure around 2.5 nC of total charge separation (left *y*-axis Fig. 5). Within the data variation bars, we do not observe any dependence of charge separation on the NaCl salt concentration. The magnitude of separated charge for all four NaCl solutions is the same as for pure water drops. Similarly, we measure ≈45 μN of average kinetic force on DI water and different NaCl solutions drops. We find that the drop friction is independent of the NaCl solution drops for the molar concentration varying in between 0.1 mM to 100 mM. Therefore, the drop discharging current, charge separation and friction force is independent of the drop's ionic conductivity in the mentioned NaCl salt concentration range. Our observation is consistent with the reports published by other authors on NaCl solutions.^52,53^

In our experiments, we do not expect a coffee ring effect, nor do we have any indication for the same. Most drops base on pure Millipore water. In case present, contamination could only be deposited at the rear side of the drop. Even for the drops with varying NaCl concentration, we do not observe any stick slip motion of the drops. We always observe a smooth motion of the rear contact line. Hence we believe that the coffee ring effect is negligible. A temperature induced Marangoni flow is another aspect. At normal lab conditions, we never detected substantial flow in drying drops. “Substantial” would be flow velocities similar to typical drop velocities of 1 mm s^−1^.

## Conclusions

The novel eDoFFI setup enables simultaneous measurements of drop discharging current and friction force acting on the sliding drops. It facilitates the direct measurement of surface charge density deposited behind the drop. With eDoFFI, we control drop speed and shape using pre-defined ring geometries. The control on drop shape provides better understanding of important physical parameters associated with the drop sliding. We find that the drop discharging current profile is similar to the drop friction, and discharging current depends on width of the drop similar to drop friction. From the delay between the drop friction and current signal at the onset of drop sliding, we conclude that the charge separation occurs only at the receding contact line. The amount of charge separated is independent of the drop speed in a low capillary number regime (≤10^−4^). With control on the drop sliding pathway *e.g.* by sliding the drop in forward and reverse motion, the neutralization time constant of surface can be directly calculated. The friction force is due the capillary force acting along the entire contact line, *i.e.* both advancing and receding contact line and charging occurs only at the receding contact line. This fundamental difference in source of origin of the force and current signal can provide better understanding on the drop stick slip motion on patterned and inhomogeneous surfaces.

## Experimental section

### Samples preparation

We prepare Trichloro (1*H*,1*H*,2*H*,2*H*-perfluorooctyl)silane (PFOTS) and trichloro(octyl)silane (OTS) on 1 mm thick Menzel glass (Thermo Fisher Scientific Ltd) by a chemical vapor deposition process.

### PFOTS deposition

As a first step, the substrate is cleansed using sonication in Milli-Q water. Sonication is performed for 10 minutes. After sonication, samples are rinsed with acetone (Sigma Aldrich, 99.5% purity) followed by ethanol absolute (Sigma Aldrich, 99.5% purity). The obtained glass slides are dried by gentle N_2_ flow. Following this step, the substrate is activated using O-plasma (chamber pressure at 0.3 mbar) for 5 min at 300 W power (Femto Diener). The activated substrate, at the end of the process, is immediately transferred to a dessicator (diameter 250 mm; Fisher Scientific GmbH). There, we expose the substrate to PFOTS vapor by pouring 1 mL of PFOTS liquid (Sigma Aldrich; CAS No. 78560-45-9) in a Petri dish in the same dessicator. Then we close the dessicator and continuously pump out the air resulting in vacuum pressure <50 mbar. The vacuum pump is run for 10 min. Then, we switched off the pump and let the samples remain in dessicator. After 20 min, the Petridish is taken out, and the vacuum is restored (<50 mbar) for another 10 min.

### OTS deposition

Substrates are cleaned and activated using the steps mentioned for PFOTS preparation. For OTS deposition, we place a Petri dish inside a dessicator and pour 200 μL of OTS liquid. Then, we close the dessicator and pump out the air till the vacuum pressure reaches <50 mbar. Once pressure reaches < 50 mbar, we shut down the pump. The substrate is exposed to OTS molecules for 120 min. After 120 min. The samples are taken out.

Before starting the experiments, samples are rinsed with ethanol (ca. 200 mL) to wash away any loosely bounded PFOTS or OTS molecules. Once the sample is placed at the measuring stage, an ionizing blower (Aerostat PC, Simco-Ion Ltd) is turned on (in cold air mode) for about 5 min directly above the sample at a height of ≈30 cm from the sample (ESI,† Fig. S9). Once the ionizing blower is stopped, we wait for another 5 min. Approximately before finally starting the current and force measurements.

After sliding a drop and performing measurements along one scan line, we re-run the ionizing blower for 5 min directly above the sample before shifting to a new scan line.

### Sensor preparation and calibration

The glass capillary (CM Scientific Ltd) is first activated with O-plasma (5 min, 300 W, 0.3 mbar). Then, the capillaries are first sputter coated (BAL-TEC MED 020 Coating system) with 5 nm thick chromium and then, with 30 nm thick gold. The obtained capillaries are then attached to a ‘BNC female jack bulk head solder mount connector’ *via* silver epoxy (MG chemicals 8330S-21G). We follow the drying procedure as stated in the manual, *i.e.* 65 °C for 120 min. After epoxy hardening, the BNC connector is connected to the input terminal of the *trans*-impedance amplifier (Femto DLPCA 200). The amplifier is fixed firmly to the vertical plate. The motion of this plate is powered by a stepper motor (KRUSS DSA 100).

The time period and spring constant of the capillary is calculated by giving the capillary an initial displacement and allowing it to undergo damping vibrations. From there, we record the time period of vibration using a CMOS camera, and use the expression: *k*_1_ = 0.24 × *m* × *ω*_*n*_^2^ to determine the spring constant. Here, *k*_1_ is the spring constant, *m* is the mass of coated capillary, and *ω*_*n*_ is the angular frequency. We estimate the spring constant in the order of 100 μN mm^−1^ for the capillary which is used to displace the small drop volumes, for example, a 7 μL drop. We used capillaries with a spring constant in the order of 500 μN mm^−1^ to displace large drop volumes, for example, 160 μL drop. To shield the sensor from the influence of external signals, we wrap aluminum foil (0.2 mm thick) in multiple folds in cylindrical form around the capillary. We ensure proper grounding of the components using a multimeter. Proper care is taken with respect to grounding of all the components.

### Force and charge measurements

For force measurements, first we capture a reference image or no force image of the capillary. This image corresponds to the situation when there is no drop attached. Then, we deposit a sessile drop on the sample and move the stage such that the drop is attached to the capillary. After that, we trigger the stage motion. The lateral adhesion of the drop to the surface causes the capillary to bend. We record the capillary motion *via* a CMOS camera throughout the drop sliding motion. The friction force acting on the drop is measured by quantifying deflection of the capillary from the reference image. The deflection (*δ*) of the sensor is measured *via* the image analysis in MATLAB 2021b. The deflection obtained is then multiplied with the spring constant to compute the friction force (eqn (8)):

8 *F*_measured_ = *k*_1_·*δ*

For the drop discharging current and charge measurements, the output terminal of the amplifier (Femto DCLPA 200) is connected to a data logger (NI USB 6009). The acquisition is triggered using a MATLAB script. Depending upon the drop speed, we vary the sampling speed. For stage speeds 0.5, 1, 2, 4, 6, 8, 10 mm s^−1^ the current data is acquired at 40, 75, 150, 280, 420, 560, 700 Hz respectively. The *trans*-impedance is kept 10^10^ V A^−1^ (rise time 50 μs) in the experiments unless stated otherwise. For the experiments with the rings, the gain is kept at 10^9^ V A^−1^. The current data is then integrated to obtain charge separated along the entire drop path. It is done by integrating the current data by trapezoidal rule with the respective sampling time fraction. To measure discharging current when the drop is shaped by different rings, we use thin silver wires (0.5 mm diameter, Chem Pure Ltd). The rings are glued to the end of conductive capillary *via* electrical conductive epoxy (RS 8330S, MG Chemical Ltd). All the measurements are performed at room temperature and at a relative humidity of 40–50%. In this relative humidity range, the slide electrification measurements are not affected by the relative humidity.^53^

### Contact angles and contact line velocity measurements

We perform image analysis using in-house developed python script to determine the sliding advancing and receding contact angles of drops, and advancing and receding contact line velocities. We take 10 pixels at each tip position and fit a tangent (image resolution: 6.25 μm per px) for the calculation of contact angles. We measure advancing and receding contact angles of 120° and 80° respectively on PFOTS coated glass, and 112° and 90° respectively on OTS coated glass. These contact angles are measured at 2 mm s^−1^ drop speed. The standard variation in the contact angles is ± 3°. To calculate the contact line velocities, we calculate the distance traversed by the drop's advancing and receding contact line in *n*th image from *n*−1th image. For this distance calculation, we consider the motion of the centroid of same 10 pixels at the drop's edges. This distance traversed is then divided by the time difference in between the each frame, which is the reciprocal of frame acquisition speed of the camera.

## Conflicts of interest

There are no conflicts to declare.

## Supplementary Material

SM-020-D4SM00019F-s001
