# Supplementary material for: Slide electrification of drops at low velocities
Source: Soft Matter. 2024 Mar 19;20(15):3349–58. doi: 10.1039/d4sm00019f (PMC11022544; doi:10.1039/d4sm00019f)
Supplement: SM-020-D4SM00019F-s001 [file SM-020-D4SM00019F-s001.pdf]

Electronic Supplementary Material (ESI) for Soft Matter.
This journal is © The Royal Society of Chemistry 2024

Supplementary Information

Slide Electrification of Drops at Low Velocities

Chirag Hinduja, Hans-Jürgen Butt, Rüdiger Berger*

Max Planck Institute for Polymer Research, 55128 Mainz, Germany

*Corresponding author; E-mail: berger@mpip-mainz.mpg.de

1. Advancing and receding contact line velocity.

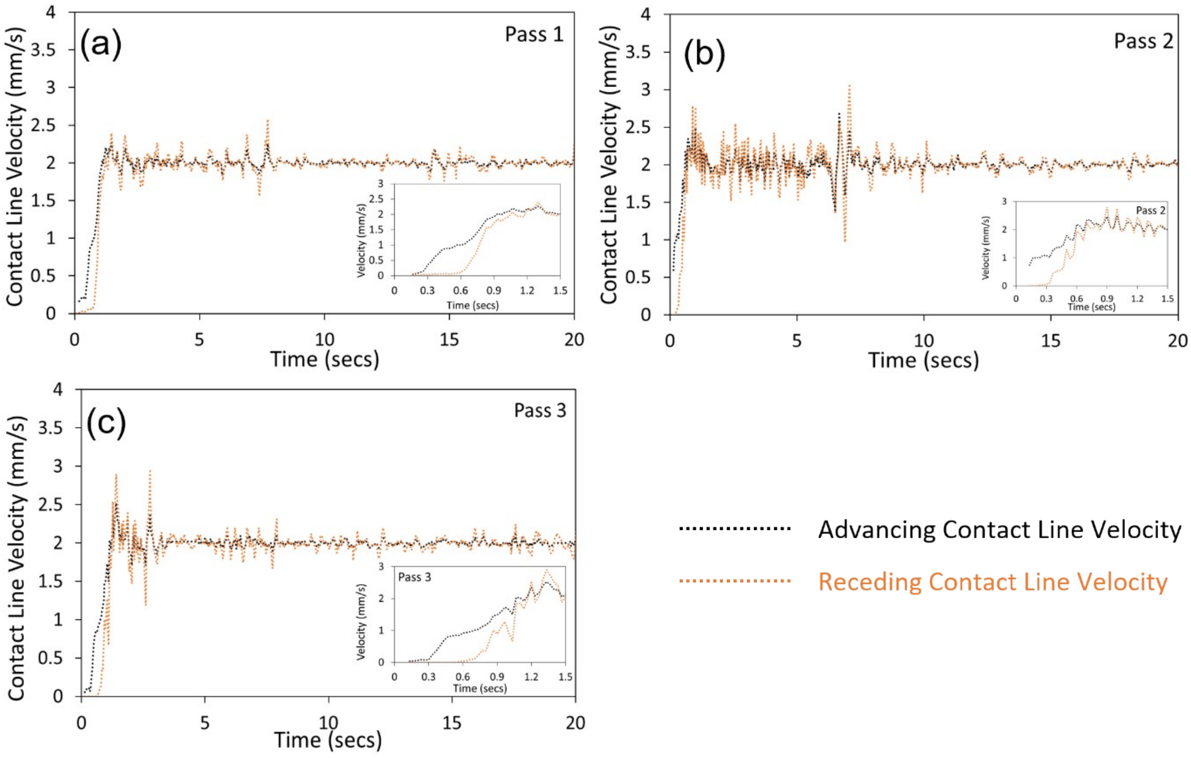

Figure S1. The advancing and receding contact line velocities for a 7 µL drop sliding at 2 mm/s speed over the distance of 40 mm. The surface is PFOTS on glass. a) The advancing and receding contact line velocities for a drop (pass 1) over a sliding distance of 40 mm. b) The velocities for 2nd drop (pass 2) c) The velocities for the 3rd drop (pass 3). The insets show the onsets of sliding for drops, respectively.

In the main section, pertaining to Figure 1, we record a current signal only when the receding contact line starts moving. We measured a current signal after approx. 0.3 seconds from the

start of advancing contact line motion. Figure S1 show velocities for the advancing and receding contact line at the onsets and during drop sliding along all three passes. For a 7 µL drop advancing contact line displaces first and receding contact line moves with a delay of approx. 0.3 seconds. The maxima in friction force and discharging current is observed when the velocity of advancing and receding contact lines are equal to the stage velocity of 2 mm/s. After that, the drop foot print rearranges to a definite shape. This rearrangement results in a decrease in friction force and indicates a transition regime. Then a constant shape of the drop with a constant foot print is reached which results in a constant current and friction signal. The contact line velocity data was smoothened by 5 points moving average for the better visualization.

2. Force and contact line velocities for each pass.

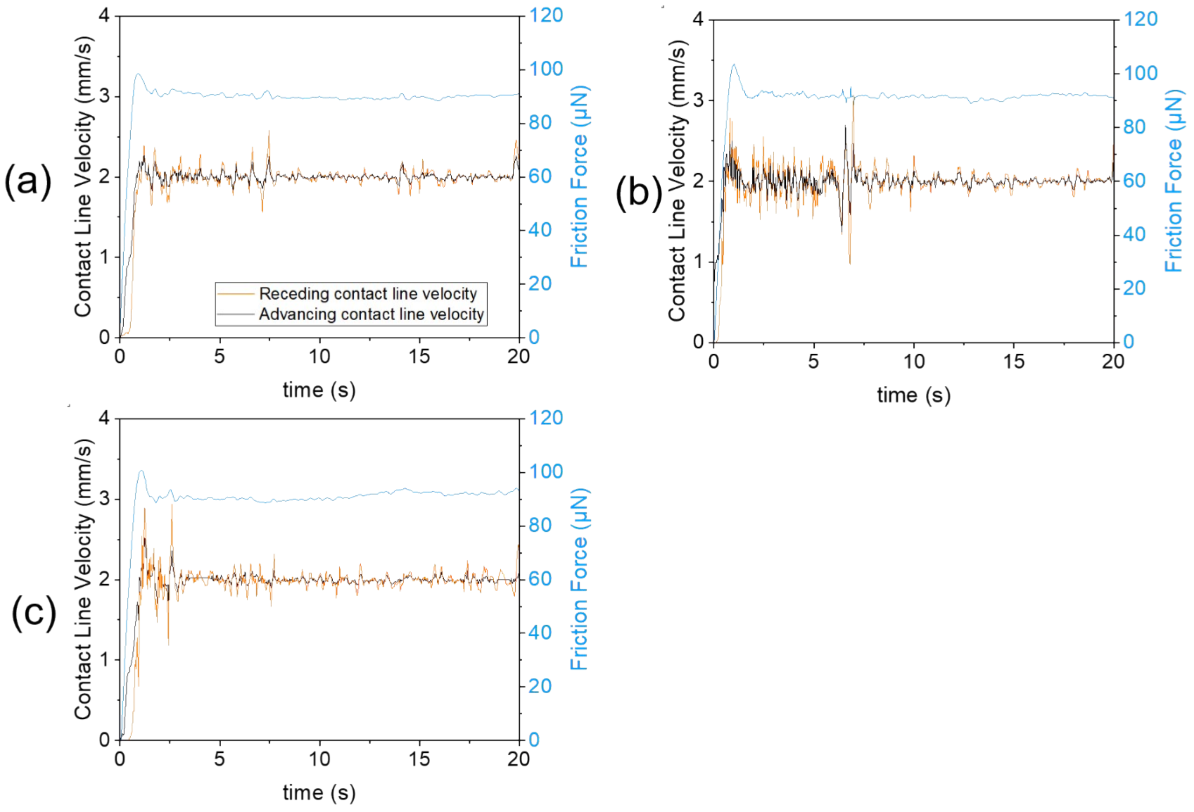

Figure S2. The friction force (blue & right y-axis), advancing (black) and receding (orange) contact line velocity (left y-axis) for each drop motion. The data corresponds to a 7 µL milli-Q water drop sliding at 2 mm/s speed on a PFOTS coated glass surface. (a) The force and contact line velocities data corresponding to the 1st drop (pass 1). (b) The force and contact line velocity data for the 2nd drop (pass 2). (c) The force and contact line velocity data for the 3rd drop (pass 3).

We plot the friction force and contact line velocities for all 3 independent drop motions (Fig S2 a,b,c). We find that the drop friction reaches maxima when the advancing and receding contact line velocity of the drop becomes equal.

3. Individual current profile.

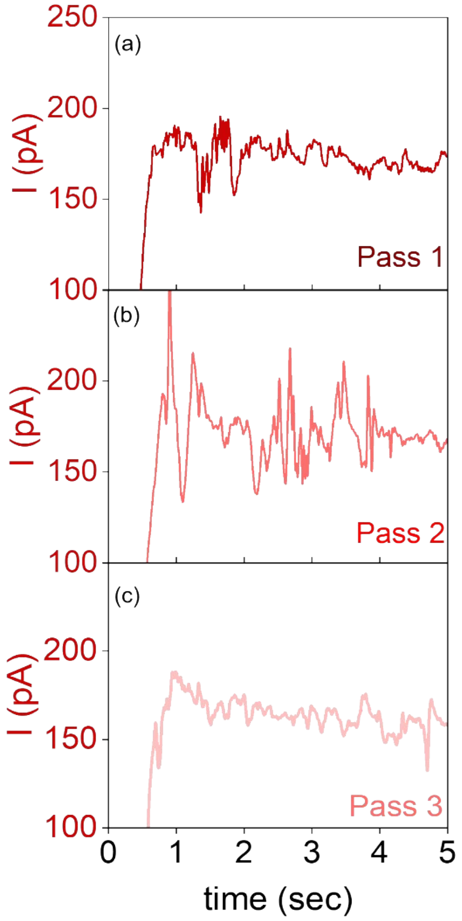

Figure S3. The drop discharging current signals at the onset of drop sliding for 7 µL water drops sliding at 2 mm/s. (a) The discharging current signal at the onset of drop sliding for the 1st drop (pass 1). (b) The discharging current signal at the onset of 2nd drop sliding (pass 2). (c) The discharging current signal at the onset of 3rd drop sliding (pass 3).

In the main text, pertaining to figure 1, along with friction force we record discharging current signals for 3 independent drop motions. To better understand and visualize the current signals at the onset of sliding, we plot individual signals against the time of drop motion (Fig S3). We observe maxima in discharging current similar to drop friction measurement.

4. Discussion on current and charge separation dependency on width.

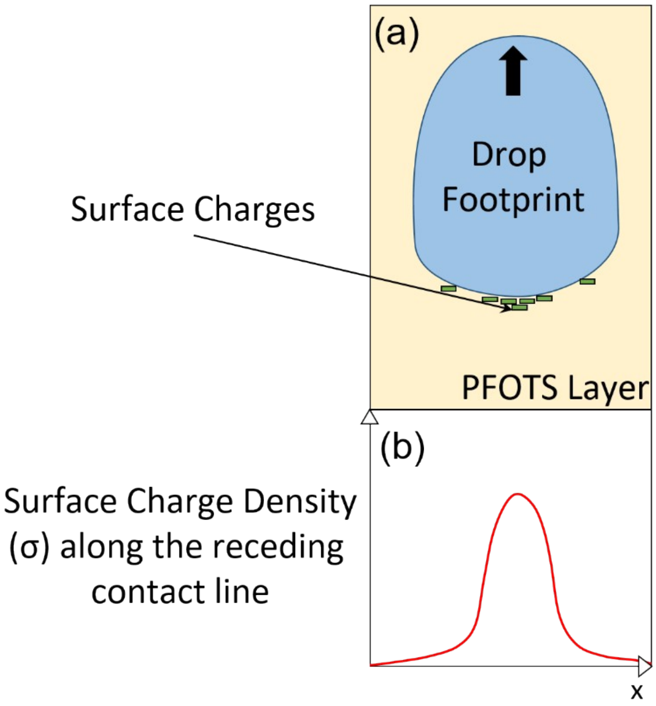

Figure S4. (a) A sketch depicting a possible distribution of negative charges (green) at the receding contact line of the drop in a top view. The sketched drop foot print resembles to the actual drop foot print when a drop slides on eDoFFI setup. (b) The aniciptaed surface charge density along the receding contact line of the drop.

In the main text, we find that the drop discharging current is proportional to width rather than drop length or the receding contact line length. It seems confusing that the charges separate at the receding contact line but it scales with drop width. We anticipate that the azimuthal contact angle distribution possibly leads to a variation in the surface charge density along the receding contact line length (Fig S4)[1].

5. Discharging current during forward and reverse sliding.

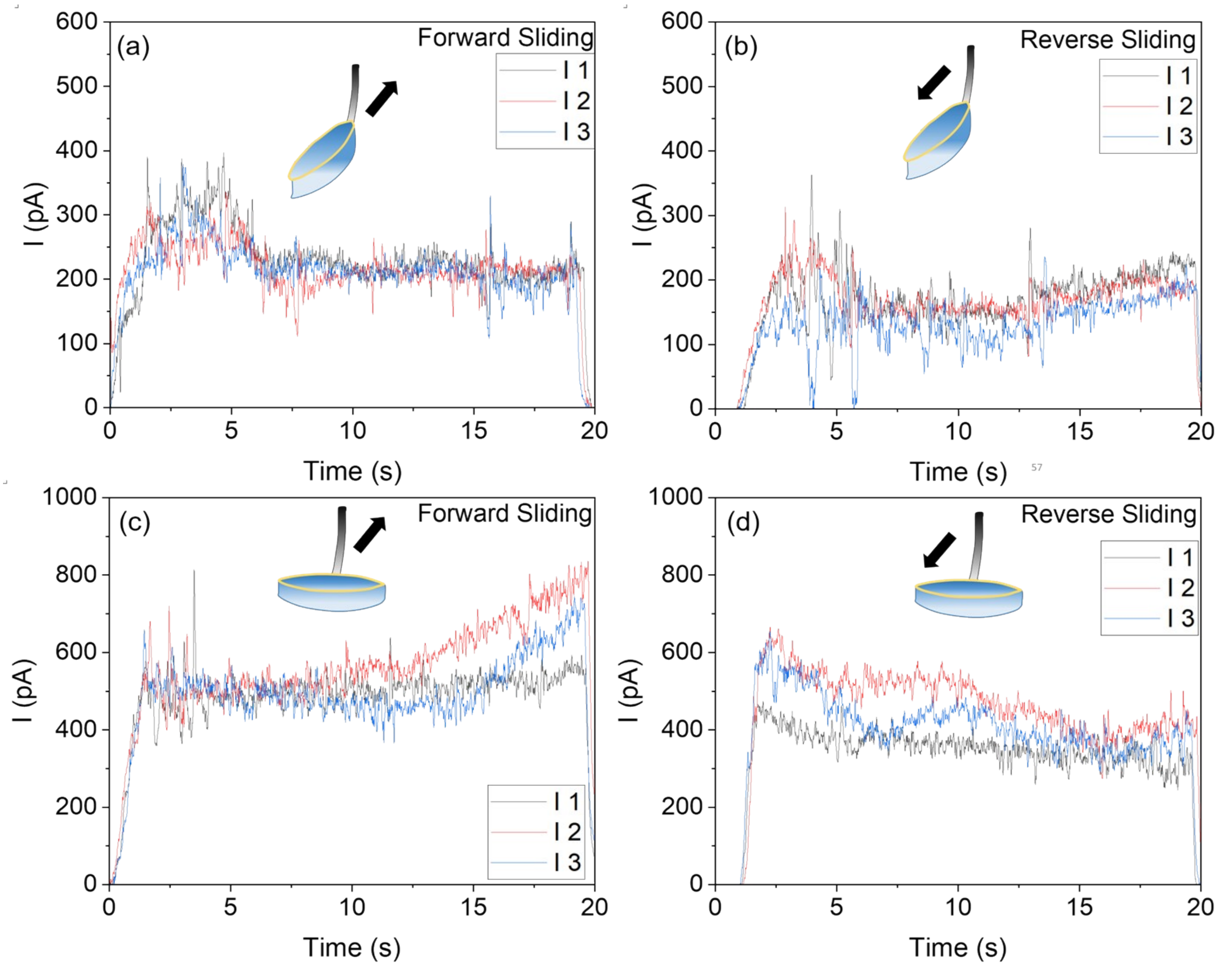

Figure S5. The discharging current signal for a 160 µL drop sliding over the distance of 40 mm on a PFOTS coated glass surface at a velocity of 2 mm/s. The current signal for (a) forward sliding with the elliptical ring which results in a larger drop length or length to width ratio of more than 1. (b) The current signal during the reverse sliding of the same drop along the respective scanline. The current signal during (c) forward sliding, and (d) reverse sliding for drops sliding with elliptical ring which results in wider drops or drop length to width ratio of less than 1. The measurements are performed on 3 independent drops using respective ring configurations. The signal is obtained at the amplification factor of 10^9 filtered with 30Hz low pass filter.

We observe a current signal of ≈ 220 pA for a drop sliding on a neutral PFOTS coated glass surface for the ring resulting in more drop length (Fig S5a). The static and transition regime of drop sliding lasts for 6-7 seconds for all 3 passes. During the reverse sliding of a drop along the respective scanline, we observe a decrease in discharging current to ≈ 160 pA (Fig S5b). The decrease in current signal is attributed to the presence of charges on the surface. These charges are deposited at the surface during the drop's forward motion. At the onset of reverse sliding,

the current signal is obtained with a delay of ≈ 1.5 seconds. This is caused by the change in direction of drop motion. The advancing contact line for the forward motion becomes the receding contact line for the reverse sliding. This change in orientation of contact angle results in a delay of ≈ 1.5 seconds.

For the ring resulting in a wider drop, we observe a current signal of ≈ 500 pA during the forward sliding (Fig S5c). We attribute the observed increase in current signal from 12 to 20 seconds of forward sliding motion to the localized surface coating variations on the PFOTS/glass surface. Due to charge deposition by the drop during forward motion, we observe a decrease in current signal ≈ 350 pA in the reverse drop sliding (Fig S5d). Again, the offset of 1.5 seconds on the time axis is observed due to the change in direction of sliding. Furthermore, from 1 to 10 seconds of drop sliding, we observe a decrease in current signal. We attribute this decrease to localized surface coating variations on the PFOTS/glass surface.

6. Additional moment on the elliptical rings.

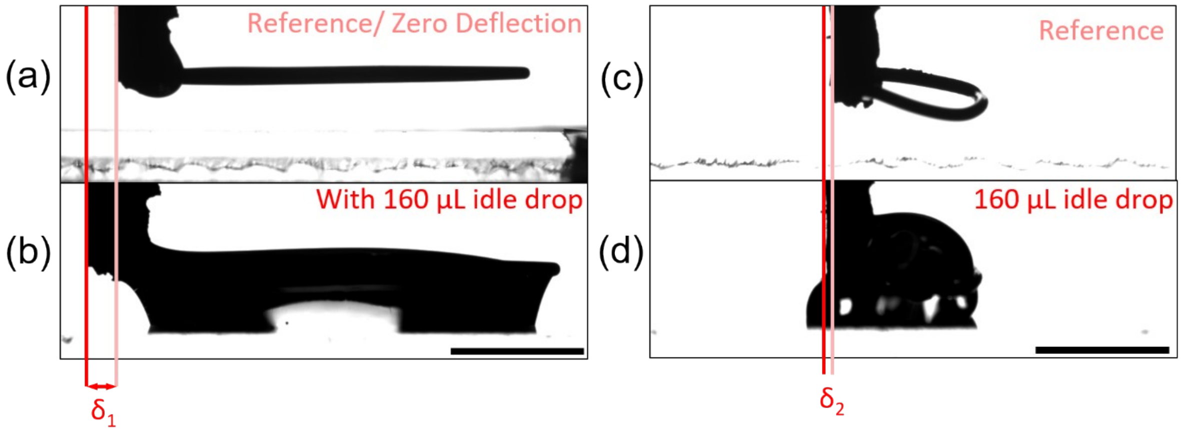

Figure S6. The images show additional torque acting on the capillary due to side attachment of the rings. The scale bars are 5 mm. (a) The reference image or no deflection image for the elliptical ring which results in lengthier drop. (b) Idle image when a 160 µL drop is attached to the ring. δ_1 is the deflection due to surface tension force on the ring. (c) Reference image for the capillary with the ring which results in wider drops. (d) Idle image when a drop of 160 µL volume is attached to the ring. δ_2 is the deflection caused by the surface tension force on the ring.

The benefit of using a side ring is that it helps us to control the drop shape. However, the attachment of the ring on the side creates an additional moment on the capillary. To measure friction force, the deflection of the sensor while the drop is sliding is measured from the

reference image. We take the reference image when no drop is attached to the rinig (Fig S6a). When a 160 μL drop is attached to the ring which results in a lengthier drop, it results in bending of the capillary, δ_1 (Fig S6b). The $\delta_1 = 1$ mm and corresponding spring constant of the capillary is 553 ± 12.5 μN/mm. This results in the force offset of ≈ 600 μN. The bending is caused by the surface tension forces acting on the ring. The net surface tension force on the ring acts at an offset from the fixed capillary axis. This offset creates an additional moment on the capillary axis. The moment results in bending which is opposite to the capillary deflection in the forward drop sliding, and it favors the deflection during reverse drop sliding. Similarly, δ_2 deflection is measured for the ring which results in wider drops (Fig S6d). The spring constant for the capillary having ring resulting in wider drops is 500 ± 11 μN/mm. Hence, we estimate a force offset of 90 μN.

7. Friction force on sliding drops with ring capillaries.

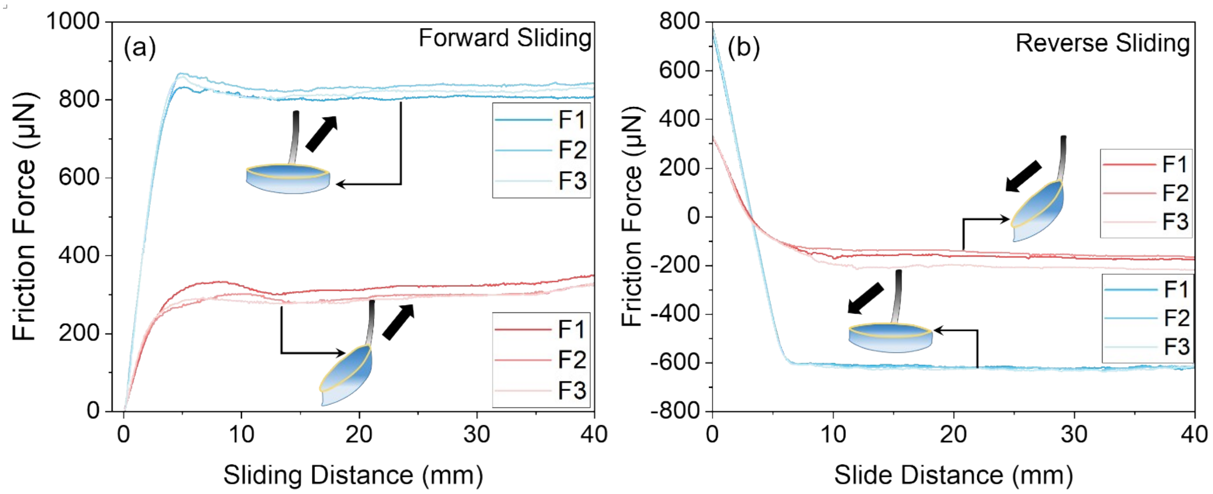

Figure S7. The friction force on a 160 μL drop displaced at 2 mm/s on a PFOTS/glass surface with both the elliptical ring capillaries. (a) The force on the drops during forward sliding. The blue data points are the forces on the drop sliding with ring which results in drop length to width ratio of less than 1. And the red data points are forces on the drops having drop length to width ratio of more than 1. (b) The force on the drops during reverse sliding. For these friction force measurements, the reference or no deflection image is when the drop is attached to the capillary (Fig S6b,d).

The actual value of friction force is altered due the presence of the additional moment (section S6). For the ring which results in lengthier drop, the presence of ring creates a force offset of 600 μN (spring $\text{constant}_1 \times \delta_1$). And for the ring which results in wider drops, we estimate a force offset of 90 μN (spring $\text{constant}_2 \times \delta_2$). To mitigate the effect of this additional moment

on our actual force measurements, we consider rings with the drop as a reference images. For instance, the image shown in figure S6b is taken as a reference image for force measurements for drop sliding with the ring resulting in lengthier drops. Similarly, the image shown in figure S6d is taken as the reference image for the force measurements for the drop sliding with the rings which results in wider drops. For forward motion using ring resulting in lengthier and wider drops, we measure force of around 810 µN and 300 µN respectively on a 160 µL drop (Fig S7a). During reverse sliding, we measure force magnitude of 170 µN and 600 µN for rings resulting in lengthier drops and wider drops respectively (Fig S7b). Laroche *et al.*, reported that by preshaping a sessile drop in a way such that the length to width ratio increases, the static drop friction can be reduced[2]. Our observation is similar to the observation made by the authors. We observe a reduction in static friction force from 800 µN to 300 µN by increasing the drop length to width ratio (Fig S7a).

8. Calculation of surface neutralization time constant.

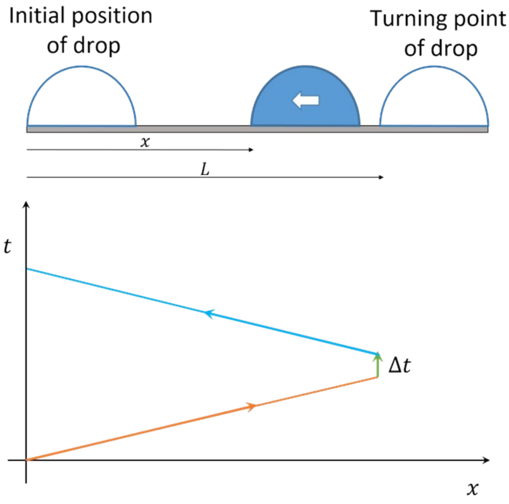

Figure S8. To calculate the time constant for the surface neutralization, we first calculate the time required by the drop to complete the drop motion along a scanline.

One of the parameters required to estimate the time constant (τ) for surface neutralization is the time, t. Where t is the time required to complete the drop motion along a scanline. The drop moves at controlled and defined velocity in eDoFFI, thus, we can convert position into time.

$$t = 2\frac{L - x}{v} + \Delta t$$

Here, Δt is the waiting time between the end of forward motion and the start of reverse motion. For a drop sliding at a constant velocity, the time t follows the schematic curve described in figure S8. The maximum time is observed at the initial position of the drop.

9. Experimental setup.

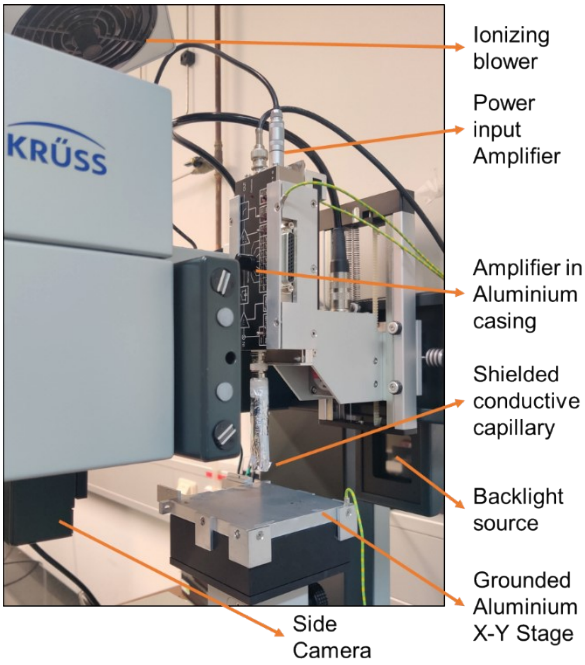

Figure S9. The experimental setup (eDoFFI) used for friction force and discharging current measurements.

The figure S9 shows the key components of eDoFFI. First, we shield the capillary by wrapping two layers of aluminium foil. This aluminium foil is connected to the ground by amplifier casing. To further shield current measurements from ambient electromagnetic disturbances, we wrap two layers of aluminium foil in the space between amplifier top and XY stage (not shown in the image). This foil acts as a faraday cage and is connected to the building ground.

References

(1) ElSherbini, A. I.; Jacobi, A. M. Retention forces and contact angles for critical liquid drops on non-horizontal surfaces. *J Colloid Interface Sci* **2006**, *299* (2), 841-849.

(2) Laroche, A.; Naga, A.; Hinduja, C.; Sharifi, A. A.; Saal, A.; Kim, H.; Gao, N.; Wooh, S.; Butt, H.-J.; Berger, R.; Vollmer, D. Tuning static drop friction. *Droplet* **2023**, *2* (1), e42.
